# Management of Recurrent Clostridium difficile Infection During Intensive Chemotherapy and Stem Cell Transplantation for Leukemia: Case with Literature Review

**DOI:** 10.7759/cureus.2413

**Published:** 2018-04-02

**Authors:** Umar Zahid, FNU Sagar, Mayar Al Mohajer, Aneela Majeed

**Affiliations:** 1 Internal Medicine, University of Arizona, Tucson, USA

**Keywords:** clostridium difficile, infection, chemotherapy, stem cell transplantation, leukemia

## Abstract

Immunocompromised patients undergoing chemotherapy for hematologic malignancy and hematopoietic stem cell transplant (HSCT) recipients are at increased risk of Clostridium difficile (C. difficile) infection (CDI). The recurrence of infection and its associated morbidity and mortality are due to multiple risk factors. Diarrhea is common in HSCT recipients, but the diagnosis of diarrhea caused by CDI is a therapeutic challenge due to frequent Clostridium difficile colonization with diarrhea secondary to non-infectious causes. The high recurrence rate is a significant challenge in the treatment of immunocompromised patients. Close monitoring of the patients, timely diagnosis, preventive measures, treatment with antibiotics, and the removal of offending agents can help in the management and cure of the disease. We review the literature on management and describe a patient with acute lymphoblastic leukemia (ALL) with multiple recurrences of CDI during leukemia therapy and allogeneic stem cell transplantation for leukemia.

## Introduction

Clostridium difficile (C. difficile) is now recognized as the most common healthcare-associated infection in the United States. Transmission may occur in the nosocomial infection setting and outcomes range from mild diarrhea to pseudomembranous colitis, toxic megacolon, and even death. C. difficile infection (CDI) is mediated by the actions of two clostridia toxins, toxin A (TcdA)  and toxin B (TcdB), which cause inflammation as well as necrosis of the colon. The risk of developing CDI is high in patients with hematologic malignancy and solid tumors and in hematopoietic stem cell transplant (HSCT) recipients due to the presence of multiple risk factors, including immunocompromised status, prolonged hospital stay during chemotherapy, and transplantation along with exposure to multiple antibiotics, chemotherapy-related disruption of the enteric mucosal barrier, the frequent use of proton pump inhibitors, histamine type 2 blockers, and gut involvement with graft versus host disease (GVHD) along with the immunosuppressive medication used to treat it [1]. Furthermore, advanced age poses an additional risk for CDI, as transplantation becomes more common in older adults. The incidence of CDI in hospitalized patients with hematological malignancies is much higher than in patients without it. The incidence of CDI following autologous and allogeneic HSCT has been reported to be 5.7% to 24.7 % during the first year after transplant [2-3]. One of the main challenges of CDI treatment in immunocompromised patients is its high rate of recurrence. Antimicrobial treatment either during or after the initial CDI episode markedly increases the risk of recurrence. With primary CDI, elderly patients and those with a severe underlying disease are at higher risk for recurrence. In HSCT, the recipient recurrence rate ranges from 2.6% to 31% [4-5].

Here, we present a case of acute lymphoblastic leukemia (ALL) with multiple recurrences of CDI during leukemia therapy and allogeneic stem cell transplantation for leukemia.

## Case presentation

A 51-year-old Caucasian female was diagnosed with biphenotypic acute lymphoblastic leukemia (ALL) in September 2015 when she presented with a two-week history of fatigue, muscle cramps, headache, cough, and weight loss. Routine blood work showed severe anemia and 25% circulating blast cells on a peripheral blood smear. A diagnostic bone marrow biopsy showed 65.1% blasts (by aspirate morphology), 90% marrow cellularity, myeloid erythroid ratio (M:E) 14.04, megakaryocytes 0.9/high power field (HPF), along with complex abnormal female cytogenetics 45, XX, del (9)(p21), -20(11)/46 XX, del(9), del(20)(q11.1). She was treated with an intensive chemotherapy ALL protocol (E1910) and achieved complete remission by day 27 of remission induction therapy. She was checked for CDI because of the complaint of fever and diarrhea and was found positive by polymerase chain reaction (PCR) of stool for NAP1/BI/027 negative C. difficile toxin within three weeks of starting therapy. The patient was treated with oral vancomycin 125 mg four times per day for a total of 10 days and repeat test was negative. The first recurrence of CDI was documented within two weeks after stopping vancomycin therapy when the patient was diagnosed with neutropenic fever, diarrhea, and a positive PCR for C. difficile with negative NAP1/BI/027. Oral vancomycin 125 mg for 14 days was resumed, which resulted in the resolution of CDI, which was evident by clinical improvement and a negative stool PCR for C. difficile toxin after therapy. During the same period, she also received posaconazole and intravenous meropenem as empiric coverage of neutropenic fever. In her first complete remission during April 2016, she received matched, unrelated donor peripheral blood stem cell transplantation with ablative conditioning (cyclophosphamide 60 mg/kg for two days and 12 Gray total body irradiation). Graft versus host disease (GVHD) prophylaxis was based on anti-thymocyte globulin, cyclosporine, and low-dose methotrexate. During the period of inpatient care for allogeneic HSCT, the patient developed a second recurrence of CDI confirmed by the presence of negative NAP1/BI/027 C. difficile toxin PCR in the stool. At this point, the patient received 10 days of fidaxomicin and then transitioned back to oral vancomycin taper therapy over the following four weeks. This resulted in complete recovery with no relapse of C. difficile. The post-transplantation evaluation after one month was negative for any morphologic or immunophenotypic evidence of leukemia and the patient had 100% donor chimerism, which is consistent with successful engraftment and leukemia remission.

## Discussion

There are many well-established risk factors for CDI during the care of patients with acute leukemia undergoing the intensive phase of chemotherapy and allogeneic HSCT. During remission induction therapy and conditioning for HSCT, the gastrointestinal tract undergoes physiological and anatomical alterations. One change is the mucosal inflammation and later ulcerations secondary to a regimen-related toxicity of intensive cytotoxic chemotherapy and another change is the suppression of marrow function in addition to a resultant alteration in the intestinal microbiota, making the intestine more susceptible to a nosocomial infection. Patients get significant neutropenia, prolonged immunosuppression, exposure to steroids, and multiple broad-spectrum antibiotics either as a prophylaxis of infections or for the treatment of neutropenic fever. Diarrhea is universally common in this patient population and is a major cause of morbidity in HSCT [6]. The etiology of diarrhea is often multifactorial in HSCT recipients and prominent causes include the adverse effects of chemotherapy, gastrointestinal graft versus host disease (GVHD) and gastrointestinal infections, while C. difficile is among the leading causes of infectious diarrhea in HSCT recipients.

Both toxigenic and non-toxigenic strains of C. difficile can colonize the gastrointestinal (GI) tract asymptomatically. Asymptomatic colonization with a toxigenic strain can proceed to symptomatic CDI. Bruminhet et al., in a recent study, reported an earlier development of CDI in patients with C. difficile colonization before HSCT in comparison to non-colonized groups [3]. Differentiating CDI in HSCT recipients from other causes of diarrhea is challenging, as diarrhea induced by other factors, such as a conditioning regimen with an asymptomatic colonization of C. difficile is difficult to distinguish from the CDI in HSCT. Akahoshi et al. reported that some percentage of patients with CDI in the early phase of HSCT may be due to regimen-related toxicity with just C. difficile colonization [7]. Although CDI may be over-diagnosed in HSCT recipients but, in clinical practice, all patients who develop diarrhea with positive C. difficile toxin are treated as CDI.

The severity of CDI is independent of the infecting strain type, but a higher 14-day mortality has been observed, with infection caused by two strains of C. difficile—NAP7-8/BK/078 and NAP1/BI/027 as well as with the presence of specific patient biomarkers: elevated white blood cell (WBC) count and C-reactive protein and low serum albumin concentration. Additionally, infection with the NAP1/BI/027 strain of C. difficile has resulted in lower cure rates and higher recurrence rates compared with other C. difficile strains when treated with either fidaxomicin or vancomycin [8].

The risk of developing CDI in allogeneic HSCT recipients is very high during the peri-transplant period, as these patients are severely neutropenic, lymphopenic, and facing the mucositogenic effects of the high dose chemotherapy or radiation and, at the same time, are receiving prophylactic broad-spectrum antimicrobials. Early diagnosis, effective treatment, and strict contact precautions are necessary for the management of CDI, but there is no recommendation to screen or treat asymptomatic HSCT recipients for a C. difficile infection; only symptomatic patients are tested and treated for CDI. Available enzyme-linked immunosorbent assay (ELIZA) assays for C. difficile toxin are not as sensitive as a cell cytotoxicity assay and does not exclude that diagnosis. Cultures increase the sensitivity further but are less specific due to the detection of the carrier and nontoxigenic strains of bacteria. PCR for CDI diagnosis is very sensitive, rapid, readily available but expensive and the major limitation is its inability to distinguish between colonization and active infection. The evaluation of glutamate dehydrogenase using an enzyme immune assay has improved sensitivity as compared to detection by latex agglutination testing but it requires pairing with one or more tests for toxigenic stain identification.

The management of CDI recurrences involves preventive measures and treatment with antibiotics along with the removal of offending agents. Recurrent CDI is a therapeutic challenge; it mostly recurs within a week but can recur up to eight weeks after the cessation of primary treatment. A CDI recurrence usually manifests as diarrhea and other signs and symptoms of CDI, and diagnosis is confirmed by the recurrence or persistence of a positive stool test. There is an increased risk of subsequent recurrence after the first recurrence of CDI. These recurrences can be with the same strain or due to a different strain.

A first CDI is treated with metronidazole or vancomycin depending upon the severity of the disease. Many patients are cured within seven to 10 days of therapy, but a longer course may be necessary for patients who are slow to respond or have a relapse. Mild to moderate disease (diarrhea with no systemic toxicities, white blood cells (WBC) < 15000 cells/mm^3^ or serum creatinine < 1.5 x baseline) is preferably treated with metronidazole, as it is cost-effective and related with reduced vancomycin-resistant enterococci (VRE) selection risk. Surawicz et al. recommend a change in therapy to vancomycin if there is no response to metronidazole within five to seven days of treatment [9]. Vancomycin is preferred over metronidazole in case of severe CDI (profuse diarrhea with symptoms of abdominal pain, sepsis, hypotension, and WBC > 15000 cells/mm^3^ or serum creatinine > 1.5 x baseline) [10], but for severe complicated CDI (characterized by shock, lactic acidosis, ileus, and/or toxic megacolon), vancomycin oral combined with metronidazole intravenous (IV) is the treatment of choice [10]. Both Infectious Diseases Society of America (IDSA) and the European Society of Clinical Microbiology and Infectious Diseases (ESCMID) guidelines recommend treatment of the first recurrence of CDI with the same therapeutic agent that was used in the initial episode [11-12], but metronidazole is not recommended by ESCMID and there has been increasing use of vancomycin in the last five years, especially for sicker patients. Fidaxomicin is an alternative agent for the treatment of the first recurrence, as ESCMID has an equal recommendation for both vancomycin and fidaxomicin in the treatment of the first recurrence [11]. Fidaxomicin is classified as a macrolide but unlike other macrolides, it is not absorbed in the gastrointestinal tract and is able to kill susceptible organisms. A second recurrence of CDI can be treated with fidaxomicin 200 mg three times daily for 10 days or by vancomycin 125 mg four times daily for 10 days followed by the pulsed or tapering regimen.

McFarland et al. reported a better cure of recurrence with the pulsed or tapered regimen of vancomycin (reported 54% of recurrence with the standard 10-14 days course of vancomycin, compared with 31% and 14% in those who had tapering regimens (gradually lowered doses) and pulsed regimens (every two to three days), respectively) [13]. There are different regimens for the tapered and/or pulsed vancomycin therapy. Surawicz et al. proposed a standard 10 days' course of 125 mg vancomycin four times a day, followed by 10 doses of pulse regimen (125 mg daily pulse every three days) [9]. Cohen et al. mentioned a different vancomycin pulsed regimen in which after the standard 10 days' course (125 mg four times a day), vancomycin is administered at 125 mg twice daily for a week, then 125 mg daily for another week, and finally 125 mg daily every two to three days for two to eight weeks [11]. There is a paucity of data on CDI treatment pertaining to hematopoietic stem cell and the solid organ transplant patient population.

Multiple studies support fecal microbiota transplantation (FMT) in the treatment of CDI in patients with recurrent disease ≥ 3 episodes after initial antibiotic therapy [14]. Cammarota et al., in a systematic review, reported that 87% of patients with recurrent CDI diarrhea treated with FMT experienced a resolution of their symptoms [15]. Despite clinical concerns, FMT is proven to be effective and safe in allogenic-HSCT and immunocompromised patients [16-17]. Probiotics are also used for the treatment of recurrent CDI, but it should not be used in transplant recipients, as these patients are generally immunosuppressed and probiotics can result in bacteremia or fungemia.

There is a paucity of data regarding C. difficile prophylaxis with antibiotics. Van Hise et al. mentioned the benefit of using oral vancomycin prophylaxis (OVP) when systemic antimicrobial therapy is required, but there is no prospective data about the OVP role in patients receiving chemotherapy. They reported a significant lower incidence of CDI in non-chemotherapy patients receiving prophylaxis with oral vancomycin (4.2% vs. 26.6%, OR 0.12, 95% CI 0.04 to 0.4, P<0.0001) [18]. However, Mullane et al. reported a significantly lower incidence of C. difficile-associated diarrhea in autologous and allogeneic HSCT patients receiving prophylaxis fidaxomicin versus placebo [19].

The patient described in our case had a recurrence of CDI during allogeneic HSCT. The first recurrence occurred within two weeks of cessation of treatment for the first episode of CDI, while the second recurrence occurred during the allogeneic transplant. The patient was treated with vancomycin for the first infection and for the first recurrence while the treatment was fidaxomicin followed by vancomycin pulsed therapy for the second recurrence, which resulted in the resolution of infection and no relapse of symptoms after stopping vancomycin.

## Conclusions

Based on the literature and guideline review, we recommend that to treat C. difficilein patients undergoing leukemia care and HSCT, they should preferably receive oral vancomycin as the first-line therapy and the same therapy during the first reoccurrence. Treatment with fidaxomicin should be considered for subsequent recurrences. There is literature to support fecal microbiota transplantation (FMT) in the treatment of CDI in patients with a recurrent disease ≥ three episodes after the failure of initial antibiotic therapy; FTM appears to be safe in the setting of immunosuppression and stem cell transplantation. Data on prophylaxis in patients receiving chemotherapy and stem cell transplant are emerging and require extensive testing in randomized prospective trials.
